# Partnership coordination for optimized COVID-19 vaccination: a case study of Benue and Niger states

**DOI:** 10.3389/fpubh.2024.1466648

**Published:** 2024-11-07

**Authors:** Saheed Dipo Isiaka, Olugbemisola Wuraola Samuel, Akolade Uthman Jimoh, Sunday Atobatele, Sidney Sampson, Victor Daniel, Joshua Cinwonsoko David, Irene Odira Okoye, Zubair Adegoke

**Affiliations:** ^1^Sydani Institute for Research and Innovation, Abuja, Nigeria; ^2^Sydani Group, Abuja, Nigeria; ^3^United States Centre for Disease Control, Atlanta, GA, United States

**Keywords:** partnership coordination, optimization, COVID-19 vaccination, case study, optimized

## Abstract

**Background:**

Developing countries have partnered with non-governmental and development organizations to ensure universal health coverage and promote equity in accessing health services. This study was motivated by the timely and relevant collaborative partnership among the National Primary Health Care Development Agency (NPHCDA), Sydani Consulting, and other implementing partners at the sub-national level. There is also no evidence of a study on partnership coordination vis-à-vis COVID-19 vaccine optimization.

**Objective:**

This study seeks to examine the influence of partnership coordination in Benue and Niger states for optimized COVID-19 vaccination.

**Methods:**

This study adopted a qualitative approach to obtain data from purposively selected participants from Benue and Niger states. Fifty-sox In-depth and Key informant interviews were conducted. The recorded discussions were transcribed and coded (inductively and deductively) using Dedoose software (v9.0). Four themes and seven sub-themes were generated from the participants' responses.

**Results:**

Findings from our study revealed that partners played significant roles in providing support to Benue and Niger states toward the optimization of COVID-19 vaccination in the two states. The provided support alleviated several challenges experienced by the states before the advent of partners in the two states. Partners' roles were assessed using the WHO Health Building Blocks Framework.

**Conclusion:**

Our study concludes and finds it plausible that partner collaboration can effectively improve health outcomes for the populace, especially in resource-low settings.

## Background

After the outbreak of COVID-19 in December 2019 (1), and its subsequent declaration as a pandemic in March 2020 (1, 2), it became imperative to achieve two objectives geared toward a fundamental goal; to control and manage the virus and improve the global population health. The first objective was to produce a vaccine that could become a panacea for the virus. This consequently emerged as a solution in the capacity of an antidote. The second objective was to embark on mass vaccination campaigns and exercises to attain herd immunity. Understanding the deadly nature of the virus, scientists worldwide embarked on creating a solution to the menace. This was achieved less than a year after the disease outbreak (3). However, fundamental concerns emerged, particularly in low and middle-income countries (LMICs), about possessing the resources and capacity to acquire and administer vaccines to their citizens (4).

To address this issue, several international organizations supported the National Health Authorities of many LMICs (5). These supports are often provided through either local organizations or the country offices of international donors. The support facilitated vaccine logistics and supply to many LMICs (6), and seldomly, these countries were also supported for vaccine administration (7). This alludes to Van Dale et al. (8) argument that intersectoral collaboration in public health strengthens health promotion, counter-acts the social determinants of health, and reduces the prevalence of chronic diseases. These collaborative efforts in the social and behavioral sciences are often referred to as *Social Capital*. Qiao et al. (9, 10) defined social capital as the network of relationships among people who live and work in a particular society enabling that society to function effectively. In other words, social capital is the collaborative efforts of a group of organizations (either within similar sectors or varying sectors) that have a shared vision of attaining similar goals.

Therefore, in the public health space, collaboration has been proven to strategically drive innovations and interventions to curb or control public health challenges and to improve health coverage within a populace (11, 12). Danahar (13) and Van Dale et al. (8) asserted that shared vision, strong relationships among partners, and leadership are prerequisites for successful collaboration. This will in turn reduce health inequalities. Several LMICs have partnered with non-governmental and development organizations to ensure universal health coverage and promote equity in accessing health services. The partnerships are established locally and/or internationally among actors in the development sector, the government, and health authorities of developing countries like Nigeria. The outcomes have yielded taking a step further in attaining Sustainable Development Goal #3 (health and wellbeing), and the 2063 Africa Agenda (health and nutrition). These efforts have also improved universal health coverage by a significant margin (14)

In March 2021, through a consortium of international donors, Nigeria received her first tranche of COVID-19 vaccines, amounting to about 4 million doses (15). The National Primary Healthcare Development Agency (NPHCDA), the leading health authority in Nigeria, initiated phased mass vaccinations across the country. This commenced with priority groups and extended to other categories of the citizenry (16). To optimally achieve herd immunity, the NPHCDA partnered with several local and international organizations in the health and social development space to provide support at the national and sub-national levels for optimizing COVID-19 vaccination. One such organization is Sydani Group. This conglomerate focuses on developing and implementing many effective sustainable policies, programmes, and practices that promote extensive development.

Sydani Consulting (a subsidiary) through the financial aid of the United States Center for Disease Control and Prevention (US-CDC), provided holistic support to Benue and Niger states. These states were selected based on their vaccination performance on the national log of COVID-19 in Nigeria. Being the two north central states in the last ten states on the list, Sydani consulting collaboratively decided with US-CDC to support the two states, particularly because of proximity for supervision. The support was provided in collaboration with other development players. These collaborators collectively pulled their resources including funds and technical expertise to support both states, complementing each other's weaknesses. Additionally, the support provided was used to strengthen the health systems of both states across the thematized World Health Organization (WHO) health building blocks.

Owing to the above, the Sydani Institute for Research and Innovation (SIRI) considered the developed partnership timely and relevant to optimizing COVID-19 vaccination in both states. Additionally, the research team was oblivious to any study that assessed partnership coordination vis-à-vis COVID-19 vaccination. In other words, we are yet to know any evidentiary information on the value of a coordinated partnership between local implementing partners and state health authorities or administrators and its influence on COVID-19 vaccination. These two factors culminated in the motivation for the conduct of the study. Therefore, this study seeks to examine the influence of partnership coordination in Benue and Niger states for optimized COVID-19 vaccination.

## Methods

This study adopted a qualitative approach to obtain data from purposively selected participants from Benue and Niger states. In-depth and Key informant interviews were conducted to elicit information from participants on Partnership coordination for optimized COVID-19 Vaccination in Benue and Niger States.

### Study settings

The study was conducted in two North Central States: Benue and Niger. One of Nigeria's six geopolitical regions, the North Central includes the majority of the Middle Belt. Seven states (Benue, Plateau, Nasarawa, Kogi, Kwara, and Niger states), including the Federal Capital Territory, make up this entity. The North Central region shares a border with Cameroon, and Benin, and spans the whole nation.

**Figure 1:**
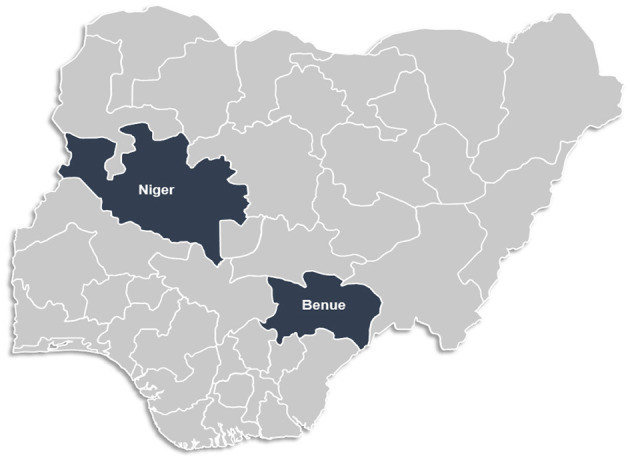
Map of Nigeria indicating the study locations.

The study was conducted in two North Central States: Benue and Niger (see Figure 1). States, Nigeria. Established from the previous Benue-Plateau State in 1976, Benue State shares international borders with Cameroon, and local borders with Nasarawa, Taraba, Kogi, Enugu, Ebonyi, Cross-Rivers, and Enugu states. The Tiv, Idoma, and Igede are the main ethnic groups that live there. Etulo, Igbo, and Jukun peoples are among the minority ethnic groups in Benue.

Niger State by landmass, is the largest state in Nigeria. It was established along with Sokoto State from the then North-Western State in 1976. The bulk of Niger State's various indigenous tribes includes the Nupe, Gbagyi, Kamuku, Kambari, Gungawa, Hun-Saare, Hausa, and Koro. The river Niger inspired the state's name.

### Study participants and selection

The study participants were made up of health workers at the local government level and the state primary healthcare level. These categories of health workers are all in the immunization unit of the primary health care agency at the state and local government levels. The health workers at the local government level include the Local Immunization Officer, the LGA M&E, and the Local Cold Chain Officer. At the state level, participants include the State Immunization Officer, the State M&E, and the State Cold Chain Officer. Subsequently, organizations partnering with the states, such as WHO, Red Cross Nigeria, UNICEF, KNVC, and Sydani Group were also involved.

A three-tier purposive selection process was adopted in identifying the relevant participants for this study. In the first tier, the two selected states were identified based on their position in the COVID-19 vaccination log in the country. The state selection was pre-determined for the research study through the design methodology of the intervention and support provided to Benue and Niger states. In the second tier, 2 LGAs were selected across three senatorial districts based on their reporting rates (high and low reporting of COVID-19 vaccination), after clustering all the LGAs in the states into three senatorial districts. LGAs with high and low reporting rates were selected (see Table 1) to explore potential peculiarities that may be associated with the selected LGAs (based on the selection criteria), which in turn may be playing a role in the level of reporting.

**Table 1:** Local government area selection based on reporting rates.

**S/N**	**Benue State**		**Niger state**	
	**High reporting**	**Low reporting**	**High reporting**	**Low reporting**
1	Makurdi	Otukpo	Mokwa	Wushishi
2	Katsina-Ala	Tarka	Chachanga	Paikoro
3	Ogbadibo	Ushongo	Edatti	Bosso

However, in this tier (second tier), two LGAs in Niger state were replaced due to security issues, resulting in one senatorial district having three LGAs, while the second senatorial district had only one LGA. In the third tier, participants were purposively selected based on their involvement in the mass vaccine administration exercise in the state.

At the time of the study, several stakeholders (across different functionality areas) were present in both states including local-level actors (state and local government areas), local implementing partners, and donors/funders. Local-level actors act as a bridge between the implementing partners or funders, and the people across communities in the state. Immunization officers manage immunization processes, monitoring and evaluation officers manage the immunization data, and the cold chain officers are responsible for vaccine storage and management. In each state, 23 local government areas were supported through the state to optimize COVID-19 vaccination. At the state level, five (5) local actors, and available implementing partners were eligible for our study. At the Local government level (LGA), three health officials in each LGA were eligible for the study, making a total of one hundred and thirty-eight (138) health officials across 46 LGAs in two states.

Participants were selected at the state level with only one criterion which was *based on their role in facilitating the vaccination exercise*. The study also purposively identified partners operating in the state and supported the state on COVID-19 vaccination and the state's health system, as key informants. The selection criteria for the partners were

(i) *provision of support to the state either in financial or technical capacity*, and
(ii) *having an established presence in the state*.

A total of 56 interviews, comprising in-depth interviews (40) and Key informant interviews (16) were conducted (Table 2). These selected participants were representatives of the total sample size as local health administrators were selected based on senatorial districts across both states, and aligns with the minimum sample size of 20 participants for qualitative interviews, while key informants were interviewed on the basis of their availability. In total, there were 52 face-to-face interviews and four virtual interviews (two in-depth interviews in Benue state, and two key informant interviews in Niger state) due to the unavailability of the participants.

**Table 2:** Participants selection in the two study locations.

**Face-to-face interview**					
**Participants**	**Niger state**		**Benue state**		**Total**
	**IDI**	**KII**	**IDI**	**KII**	
**Local government-level actors**					
Local Government Immunization Officer	6		5		11
Local Government M&E Officer	6		4		10
Local Government Cold Chain Officer	6		5		11
**State—level actors**					
PM SERRIC	1				1
State Immunization Officer	1				1
State Health Education Officer	1				1
State M&E Officer	1				1
State Assistant Director for Logistics			1		1
State Cold Chain Officer	1				1
**Other implementing partners**					
Sydani Group		4		4	8
Red Cross Nigeria				2	2
Heartland Alliance		1			1
Breakthrough Action Nigeria				1	1
**Donors/funders**					
WHO				1	1
UNICEF1				1	1
**Virtual Interview**					
State Executive Secretary			1		1
State Health Education Officer			1		1
Palladium (Local Partner)		1			1
Chigari Foundation (Local Partner)		1			1
**Total**					**56**

### Inclusion criteria for selecting study participants

The local-level actors were selected based on the following conditions

(i) Engaged in their respective positions and roles by Sydani consulting and other implementing partners to facilitate the optimization of the COVID-19 vaccination exercise.

(ii) They must have also visited the LGAs, wards, facilities, and communities to supervise and observe vaccination exercises.

(iii) They must have been doing this for over three (3) months.

### Exclusion criteria for selecting study participants

On the other hand, participants were excluded if they were

(i) Either health workers at the grassroots level (facility and outreach teams)
(ii) Did not participate in the COVID-19 vaccination exercise.

### Analytical framework

According to the WHO, Health systems consist of organizations, people, and actions to promote, restore, or maintain health (17, 18). WHO emphasizes that the health system is a set of interconnected parts that must function together to be effective, and as a result proposed the six health system building blocks. Managing the interaction among these blocks translates to achieving more equitable and sustained improvements across health services and health outcomes.

**Figure 2:**
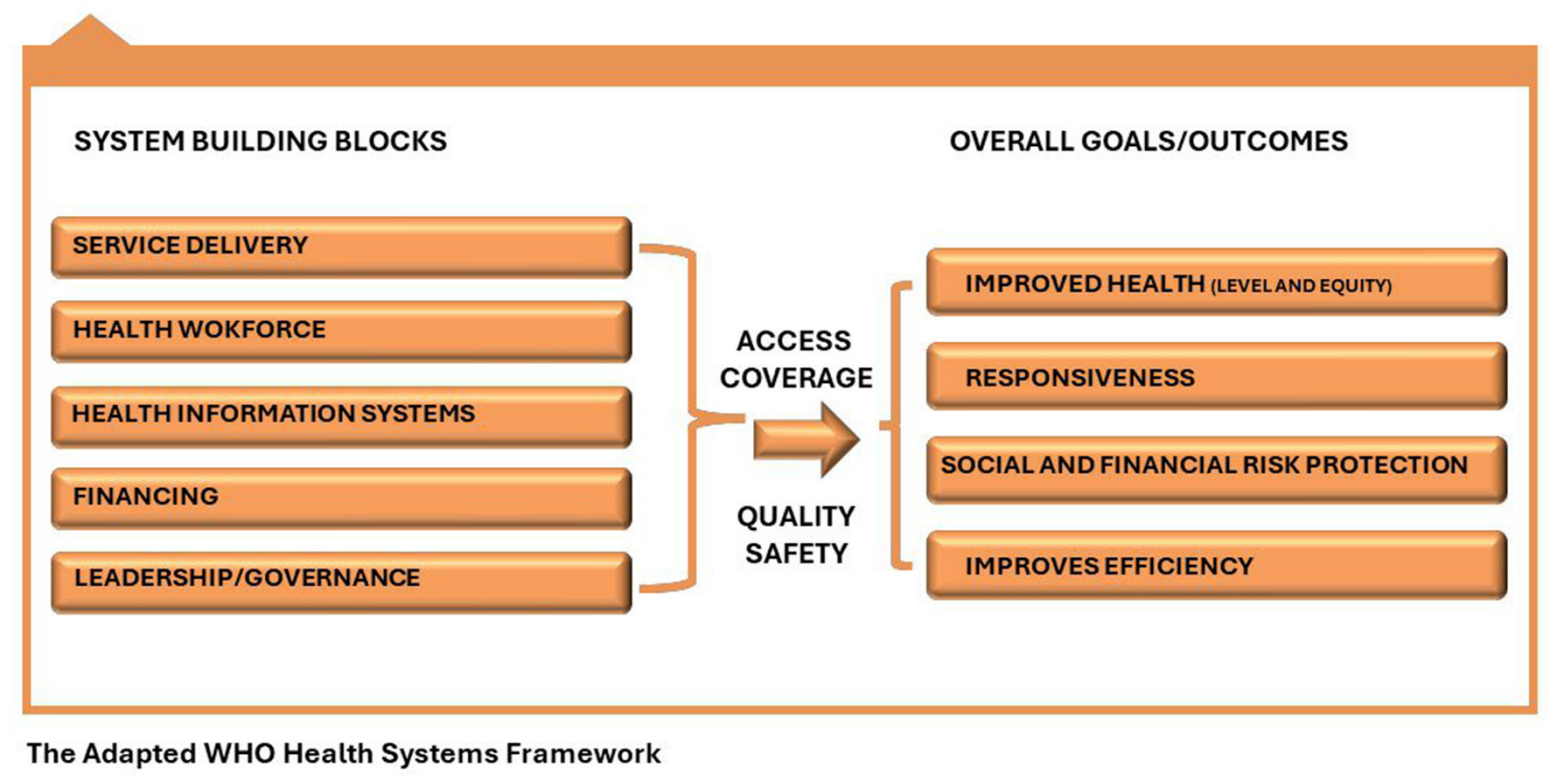
The adapted WHO health system framework.

The WHO Health System Building Framework was adapted in this study to scientifically and constructively guide the study tool design, data collection, and analysis (17). The framework has six components and five were adapted for this study (see Figure 2) as they are relevant to guiding the operationalization of our objectives.

### Ethical approval and consent to participate

The research protocols were reviewed and approved by the National Emergency Routine Immunization Coordinating Centre (NERICC), the Institutional (internal) Review Board of the National Primary Health Care Development Agency (NPHCDA) in Abuja, Nigeria. The research adhered to the principles outlined in the Helsinki Declaration. Additionally, written informed consent was obtained from each participant prior to the commencement of the research.

### Data collection

A semi-structured interview guide was developed for each category of respondents using the WHO Health System Building Blocks Framework (17). Specifically, the study assessed the role of implementing partners and/or funders across service delivery, health workforce, health information systems, finance, vaccine logistics, and leadership. These areas were focused on to document the influence of implementing partners' support on the health system of the supported states. Questions were generated for these thematic areas based on the sole objective of the study, and the identified areas best apply to them. Questions such as: “Kindly describe the roles of partners in the following areas; service delivery, health workforce, health information systems…. (within the immunization space) during the last two years” and “Generally, describe the roles your organization played in supporting the state to optimize COVID-19 vaccination within the state in the last 2 years”.

Fifty-six (56) interviews; comprising in-depth interviews (40) and Key informant interviews (16) were conducted among local actors and implementing partners in Benue (26 interviews) and Niger (30 interviews) states. Two (one male and one female) professionally trained researchers working with SIRI were responsible for conducting the interviews. Aside from reaching out to the study participants during the recruitment phase via mail sent to the two states through Sydani Consulting, no form of contact was established between the researchers and the study participants. Verbal consent was obtained from all the participants before the interviews were conducted, by asking the participants if they were willing to participate in the study and accept that the discussion be recorded. All discussions were conducted in English language to ease communication within 30–60 min on an audio recording device.

### Data analysis

All audio recordings were transcribed in Word document files by professional and experienced transcribers. The research team reviewed the transcripts' contents and developed the codebook with the adapted WHO health building blocks framework (17). The transcripts were coded and managed on Dedoose software (Version 9.0) combining the inductive and deductive approaches (19). A thematic analysis was conducted, and codes were synthesized to identify useful patterns in responding to the study objective. The excerpts were reviewed to make connections between and within participants' responses to seek explanations for the patterns of the themes. Additionally, transcripts were randomly reviewed by the research lead to observe alignment in the themes and sub-themes generated from the participants' responses. Three thematic areas were generated from the study including an overview of the COVID-19 vaccination exercise, partners' roles in optimizing COVID-19 vaccination in Benue and Niger states, and participants' perception toward partners' support in Benue and Niger states.

## Results

**Figure 3:**
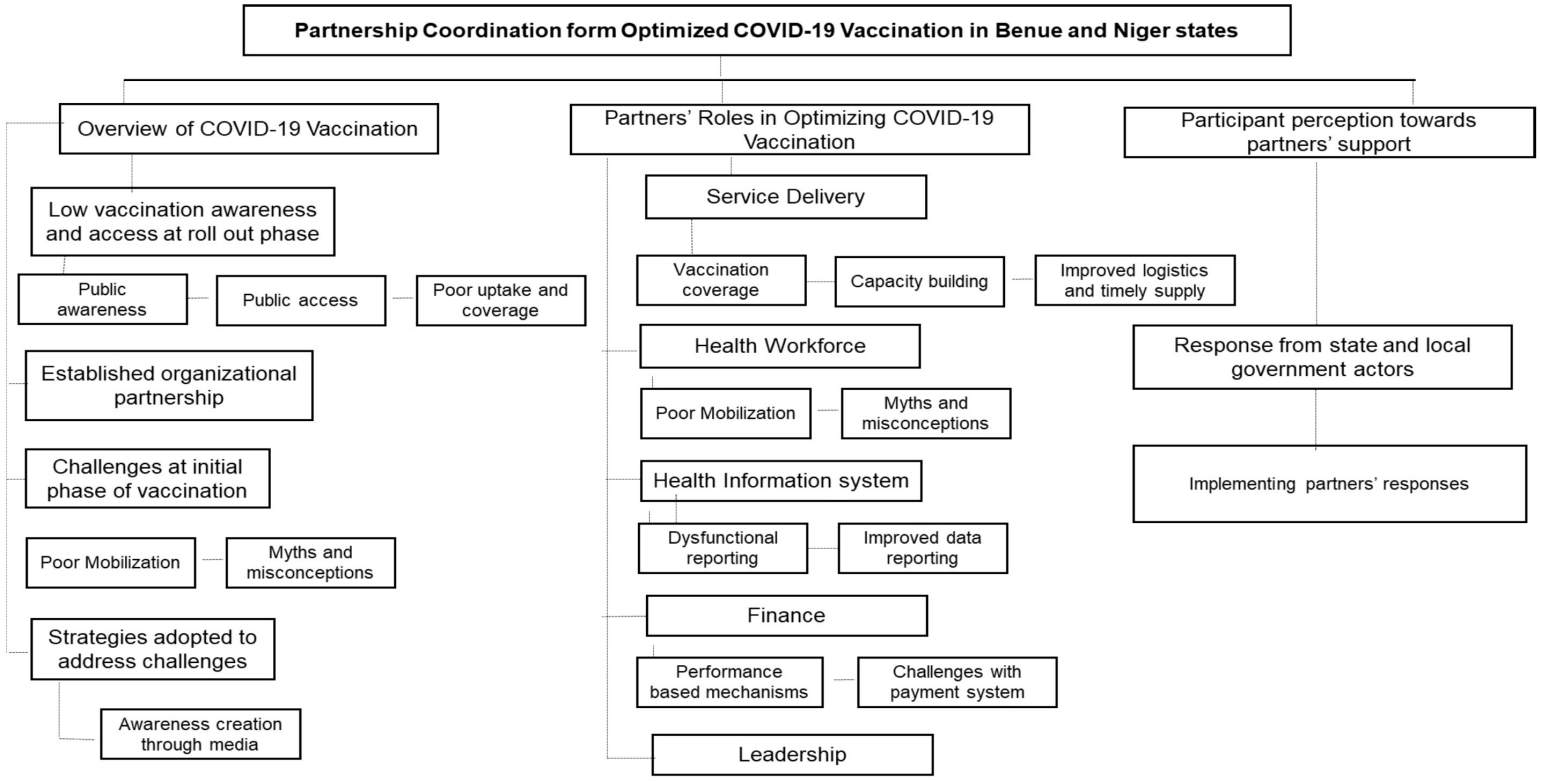
Thematic analysis of study findings (themes and sub-themes).

This study explored partners' contribution to optimizing COVID-19 vaccination in Benue and Niger states, as well as the roles of stakeholders in strengthening the health system in both states using In-depth interviews and Key informant interviews. The following three major areas evolved from the thematic analysis which speaks to (i) understanding the COVID-19 vaccination exercise over the last 2 years (2021–2023), (ii) the roles partners played in the optimization of COVID-19 vaccination and meeting the national target in the states across 5 key areas influenced, and (iii) perception of stakeholders at the state and LGA levels on the support provided to the states by partners (see Figure 3).

### Socio-demographic characteristics of participants

We interviewed 56 participants across Benue and Niger states. The majority (78.6%) of the study participants were male by gender, and a little above half (57.1%) were local government health officials (PHC Director, LIO, LHEO, M&E, and CCO). The mean age of the respondent is 44.2 years while about half (51.8%) of the participants have a university degree (Table 3).

**Table 3:** Socio-demographic characteristics of participants.

	**Niger state**		**benue state**	
**Characteristics**	**Frequency**	**Percent**	**Frequency**	**Percent**
**Gender**				
Male	25	83.3	19	73.1
Female	5	16.7	7	26.9
**Age group**				
30–39	8	26.7	9	34.6
40–49	15	50	11	42.3
50–59	6	20	4	15.4
60–63	1	3.3	2	7.7
**Level of education**				
Diploma	11	36.7	5	19.3
NCE	5	16.7	2	7.7
BSc	13	43.3	16	61.5
Masters	1	3.3	3	11.5
Total	30	100	26	100

### Overview of COVID-19 vaccination exercise

#### Low COVID-19 vaccination awareness and access at the roll-out phase

To understand the general state of the COVID-19 vaccination exercise in both states, participants interviewed at the state and LGA levels were requested to describe their experience from the roll-out phase of the vaccine till the time of the study. All the participants expressed their challenges across three sub-themes: **awareness, accessibility, uptake**, and **coverage**. Based on this, participants expressed that the level of COVID-19 vaccination awareness among residents in their respective LGAs and states was extremely low throughout the roll-out phase of the vaccine mainly due to high levels of misconception.

##### Public awareness

Subsequently, the participants emphasized that the limited awareness of COVID-19 vaccines and vaccination that existed in their respective states to some extent, played a role in the relative accessibility of the populace to the vaccines in the two states, thereby resulting in limited coverage of COVID-19 vaccination. However, there was a positive shift over time, after efforts were made to address all necessary concerns. This is evident in the expressions of the study participants.

*Initially, as the program started, people shied away from the vaccination. They were even saying that this was just a rumor. There was nothing really like COVID-19, until when we started the awareness campaigns'and then we started making some consultations, involving people like the traditional leaders and the religious leaders, they knew that COVID-19 was truly in existence. So, people started coming out, and when they started getting the vaccines, especially the AstraZeneca when it comes, people were very interested* ***(IDI/LCCO/Katsina Alana/Benue*** ***State)***.

*Well, initially I would say the awareness wasn't as much as now because when it all started in 2021, it began gradually, with the creation of a task force using the ACSMs and media houses, the awareness became more vast and people now understood the effects, the challenges, and also the side effects around not being vaccinated with COVID-19 vaccines. So, the awareness became broad and people, and everybody now understood that there is a need for them to take COVID-19 vaccination* ***(IDI/State M&E Officer/Niger State)***.

##### Public access

Additionally, participants equally revealed that there were variations in the accessibility of the COVID-19 vaccine in their various domains but highlighted that there was increased accessibility over time.

*No, there are ways people have been accessing the vaccination from 2021 till now, they have been accessing it, but the trend is high now compared to previous times. Why is it so? In the beginning, there was no demand creation for vaccines, and even now the jingle has stopped. It used to be partners funding the creation of adverts, television, and all those things. It has reduced so people now say that the politicians have come in and are getting what they want. They have left the team to do it alone* ***(IDI/LIO/Tarka LGA/Benue*** ***State)***.

*Ok you know at the beginning; accessibility and the number of teams were not much like it is presently. when we started SCALES 3.0 we started with 6 teams in the whole LGA Gabadaya, but presently, we have more than 40 teams in the LGA. In almost every ward you will see more than 10 vaccination sites very close to the community or the people in the settlement so the accessibility is very good. There's no issue in terms of accessibility or accessing the processing points* ***(IDI/LIO/Chachanga LGA/Niger State)***.

##### Poor COVID−19 vaccination uptake and low coverage

Coincidentally, discussions from participants revealed that at the beginning of the COVID-19 vaccination exercise in Benue and Niger states, there was a connection between the populace's limited awareness, and their uptake and subsequently the coverage of the vaccination across both states. This was exuded in the following expressions:

*Yes, I must say that coverage was initially low because there were a lot of misconceptions and rumors at the beginning but with the help of awareness creation, as I just mentioned, the people could eventually access the vaccine on site and the discovery was very significant as I earlier stated. Despite our target initially not meeting the national expectation, we still try to push because people can be difficult to change in behavior. Regardless, we made sure we still use all our mechanisms to mobilize our people to sensitize them (**IDI/State Health Education Officer/Niger*** ***State**)*.

*For coverage when we started you know we've taken several approaches in this coverage. We started with phase one; then we now go to SCALES 1.0, 2.0, and then to 3.0. So, for each of the strategies, there were some lessons learned and what I'll say is that when we started, the coverage was actually nothing to write about and this has to do with some challenges like human resources and logistics that were poorly available. Though, with the support of the partners, we were now able to make changes and vaccinate more people for COVID-19 in my state (**IDI/State Immunization Officer/Niger State**)*.

Discussions with the study participants indicated that initial efforts from the local actors toward COVID-19 vaccination were not yielding the expected result, and this was premised on some issues including human resources, and logistics (particularly for vaccine transportation), among others.

#### Established organizational partnership

Findings from the study revealed that the Nigerian government, international donor organizations such as the World Health Organization (WHO) and the United Nations International Children Emergency Fund (UNICEF), as well as local implementing partner organizations such as RISE and Sydani Group (consulting) provided financial and/or technical support to Benue and Niger states across two phases. However, Sydani Consulting provided this support across two phases of the project. Participants emphasized that they received support from multiple organizations over the past two (2) years to drive the optimization of COVID-19 vaccination. Participants also elucidated that the Sydani provided support consistently.

*The partner that first did this support was WHO. Then after WHO Sydani came in and also supported. There was a team called the RISE team, which also gave their support then at the end of it we had one Heartland alliance. They also contributed to the implementation of COVID*−*19 and the support of SYDANI and RISE team, Heartland alliance, and WHO support us in Bosso LG. I will say the entire Bosso local government. Because the teams were no longer being paid so they were completely discouraged. But with the support of those organizations, they were motivated to work* ***(IDI/Cold Chain Officer/Bosso LGA/Niger*** ***State**)*.

*To be sincere, the partners, especially the Sydani group, really tried. If not for that group, people wouldn't have been vaccinated up to 20%. But since they came up with all the strategies they put in place, people started accepting these services and they are coming up I am speaking from the report I am getting. Even in the interior part of this local government, we thought people would not even like to be vaccinated, but people accepted it. Then, we also have support from KNCV and Breakthrough Action* ***(IDI/M&E Officer/Ogbadibo*** ***LGA/Benue State**)*.

#### Challenges experienced by states and LGAs during the initial phase of COVID-19 vaccine optimization

##### Poor mobilization

While Benue and Niger states commenced the administration of COVID-19 vaccination to their respective residents, they were both confronted with several challenges that affected their vaccination process. One of these challenges was the poor mobilization of health workers. Findings from the participants shed more on this:

*We encountered challenges because it was only when the health workers got paid, that they were motivated to work, but when they started work, they were not paid, and a lot of people were working at the time, especially during SCALES 3.0, and they still did not pay them. So, all the health workers withdrew and did not participate anymore* ***(IDI/State Cold Chain Officer/Niger*** ***State**)*.

*When the COVID-19 activity started initially, there was no mass information, there was no mobilization or any sensitization about it. Health workers were ready to work but there was nothing to encourage them. Even, when we told them to please go, they would tell us that “Are we not going to eat?”* ***(IDI/M&E Officer/Ogbadibo/Benue State**)*.

##### Myths and misconceptions

The second challenge was misconceptions among residents of both states. Myths and misconceptions about the COVID−19 vaccine were the strongholds that prevented many persons residing in the study areas from being vaccinated. This also frustrated the efforts of the vaccination team and other stakeholders during the initial phase of the vaccine optimization.

*The only problem we faced initially, that lingered for some time before it finally resolved itself is this resistance to the uptake of this vaccination because of all these fake news, and rumors about the vaccinations that people are hearing about. That one went viral than the real message that we were initially giving* ***(IDI/M&E Officer/Ogbadibo/Benue*** ***State**)*.

*Initially, our people were saying “That thing is a white man's disease, not a black man like us”. So, they refused to take the vaccine but subsequently, most of our people fell victim to the disease* ***(IDI/LIO/Ushongo/Benue State**)*.

*I think I told you that in the beginning, people termed it to be the vaccine that would harm them and said “Allah Kiya yae” (I will not take the vaccine) because it happened to so… so… so… person. They were even showing some pictures that when people do take the injection, it will harm them, and their blood will clot and it would lead to death before 6 months, that people will die* ***(IDI/M&E Officer/Wushishi/Niger State**)*.

#### Strategies adopted by implementing partners in addressing the challenges

Consequently, participants were requested to describe the partners' approach to addressing the extant problems that befell the states during the administration of COVID-19 vaccination. Responses from participants revealed that the partners played a significant role in addressing their extant challenges, and this also eased the implementation of intervention on the part of the partners.

##### Awareness creation through different media platforms

*So, when all these partners came, we came as a group to discuss our challenges, and what we could learn from the previous phases of the COVID-19 vaccination and the major thing which was the biggest challenge was transportation logistics for the teams, and when the partners came those were the targeted areas. They supported the teams so that we could access the hard-to-reach communities and scattered settlements. Also, the partners were able to convince the villagers of the existence of the COVID*−*19 virus and that they should be vaccinated. Partners have been of great help, they have supported through advocacy, communication, and social mobilization creating awareness. Some of them go to media houses to have a radio discussion to sensitize the people about COVID* −*19 vaccine and this helped to address most of these challenges (**IDI/State Immunization*** ***Officer/Niger State**)*.

*When partners such as Sydani came, they ensured that our challenges at the state level, especially at the management and coordination level, were addressed. The partners helped us create awareness. For instance, Sydani and RISE supported our radio jingles for a few months, and they helped our people very well to know about the vaccine (**IDI/PM*** ***SERRIC/Niger State)***.

Essentially, partners were able to address the extant challenges identified in the states. However, evidence revealed that partners dialogued with the state health authorities on their arrival in both states to discuss pertinent issues peculiar to COVID-19 vaccination being experienced within the state when administering COVID-19 vaccination to their respective populace.

### Partners' roles in optimizing COVID-19 vaccination in Benue and Niger states

Interestingly, findings from the discussion with participants indicated that the COVID-19 vaccination exercise in Benue and Niger states commenced with support from the National level through vaccine logistics to the states. However, partners played key roles in driving the optimization of the vaccination in several settlements within the LGAs in the states. Utilizing the WHO health system building blocks, we presented the partners' support in the state across the following health system blocks: Service delivery, Health Workforce, Health Information System, Finance, and Leadership.

#### Service delivery

To assess partners' roles in driving service delivery, participants were requested to discuss partners' contributions to vaccination coverage, vaccine safety, and vaccine accessibility. This breakdown was essential to explore the nitty-gritty of partners' support toward standard service delivery in the two selected states.

##### Vaccination coverage

All interviewed participants at the state and LGA levels reported that partners financially supported the state to drive the optimization of vaccination, subsequently increasing the coverage. Partners also ensured accessibility through vaccine logistics, and Solar Direct Drive (SDD) Refrigerators provided by partners ensured vaccine safety.

*The coverage is increasing if you compare when we started, to this point in time, that it is up to 60% coverage., or more. The range (of over 60%) covers the targeted people who have already been vaccinated. Most of them are on booster doses. Yes, I must say that, if not for partners, it would have been impossible to cover over 60%, because the government was not ready to fund it. The partners meant business and were even responsible for the movement of vaccines. The partners play a major role* ***(IDI/Cold Chain Officer/Otukpo LGA/Benue State**)*.

##### Capacity building through training and integrated supervision

The study findings revealed that participants eulogized the support the partners provided to them in ensuring that they were equipped with the required “technical know-how” that is expected of the actors at the state and LGA levels to ensure seamless service delivery to residents of the states, translating into an improved vaccination coverage.

*Yes, partners played a great role. There was training across all the levels in the state because as RISE was training, Sydani too was training. They trained people at the facility, the LGA. The state-level training was for supervision, particularly on using the ODK tool. Periodically, we do receive messages, we have their contacts, and we do have Zoom meetings, during this time when there are questions, errors, misunderstandings, and misconceptions all these things would now be trashed. During supportive supervision, health workers acted as our participants, and we corrected where necessary. Partners played a great role in building a knowledgeable and strong team, that worked well. And that helped us in coverage and data transmission (**IDI/LGA Immunization Officer/Bosso LGA/Niger*** ***State**)*.

*All the partners contributed to capacity-building, I think you understand- training of our health workers. It was the WHO, Sydani, and RISE that trained all of us very well, and even my LIO went for the training so that he can be part of the team that will conduct supportive supervision* ***(IDI/LGA M&E Officer/Bosso LGA/Niger State**)*.

##### Improved logistics and timely supply of vaccines

Participants enunciated that partners significantly facilitated the logistics of COVID-19 vaccines across the various levels of vaccine logistics starting from the national cold store down to the facilities where the vaccines were temporarily stored for use and being stored permanently at the state and LGA levels for easy accessibility in a time of need.

*There is a good system of vaccine logistics and supply in the state. First, the National Primary Healthcare Development Agency helps us to send vaccines to the states, and I am aware that they partnered with UNICEF to move the vaccines here. At the state level, our partners like Sydani Group or Breakthrough Action and Red Cross Nigeria help us to move vaccines to the LGAs, though only Sydani either provide funds or move vaccines to many of the LGAs* ***(IDI/Assistant Logistics Director/Benue*** ***State)***.

*Aside from other partners involved in COVID-19, GAVI played a very important role. They supplied solar refrigerators to our local government offices and facilities. As we speak now, all the headquarters have solar refrigerators supplied by GAVI and that is where we keep these vaccines. You see, that is where we keep these vaccines and without that, in those days, we only have one center here, where we store our vaccines and supply them to individuals at their doorstep within the ward here, so there's no need to travel on daily basis to give them (**IDI/LGA Immunization Officer/Otukpo/Benue State)***.

The key informants highlighted how their strategic approach of information gathering and the use of technological tools such as Geographic Information System (GIS) aided their development of a comprehensive strategy to facilitate a structured service delivery method geared toward the goal of increasing the reach of COVID-19 vaccine in the various settlements across the two states, ensuring further steps are taken toward the attainment of national target as required.

*Ok, just as I mentioned earlier, we provide technical support in COVID*−*19 vaccine activities however in service delivery we have been able to support, provide technical support to the logistics team by providing information, getting the data from the local government, getting stock balance and also preparing distribution plan and helping the team, the state code chain officer, bringing information to the state Code officer, highlighting the local government with stock that needs to be supply and also identifying key stakeholders that the state can follow up at the national level to also move for supply of vaccine so they can deliver to all the local governments (**KII/Strategic Information*** ***Associate/Palladium)***.

*Service delivery was a major or was made up of multiple efforts. There were so many things we strategized, or tried, some of which included GIS mapping. We deployed in two places that were densely populated and had unvaccinated people. We deployed teams to markets, churches, mosques, high profile areas that would have crowds gathered on specific days (**KII/Senior Analyst/Sydani Group**)*.

#### Health workforce

For the health workforce, findings from interviews with stakeholders at different levels revealed that before the arrival of the partners in the state, there were two major concerns associated with the health workforce of both states. Fundamentally, the health workers were not being financially motivated sufficiently, based on the perception of state officials and some of the LGA officials.

Secondly, some LGA officials held the perception that the health workforce is not entirely adequate, due to increased attrition rate, unemployment, and the volume of available tasks for health workers, particularly in the rural LGAs to drive vaccination at the grassroots level.

(a) *Health workforce challenges*

*Lack of incentives and motivation*

Interestingly, participants emphasized that as health workers geared toward ensuring the optimization of COVID-19 vaccination in the rural communities of both Benue and Niger states, they experienced issues around extrinsic motivation, particularly concerning finances that not only spurred them to work but also supported their various activities, such as transportation to the several targeted communities.

*So, when we started the big challenge was the fund that would be given to these guys because when we started it was just like a reimbursement process. Reimbursement simply means that the teams were going to work for a certain period and after the satisfaction of the organization then they'll now come and pay the health care workers. So as time went on a lot of issues occurred in the form of payment of logistics and payment of stipends for the participants. Most of those who were expected to go for outreaches actually remained in their various house because of lack of payment* ***(IDI/State Immunization Officer/Niger*** ***State**)*.

*So, before we used to have several teams that worked for us at the fixed post and the outreach teams, but because the government was not paying them very well for the work they were doing, many people decided to sit at home and not go to work, and you cannot blame them. They also want to eat and feed their families (**IDI/LGA Cold Chain*** ***Officer/Ogbadibo/Benue State)***.

*Excess workload*

Essentially, participants expressed concerns over having too much to do, influenced by the shortage of health workers at the facility level, and the government's inability to recruit more health workers. According to the participants, they were generally overwhelmed with having to take on multiple responsibilities to ensure appropriate service delivery.

*Seriously there were changes during the COVID-19 period because we didn't have any instruction that we should recruit a volunteer who would support us in terms of vaccinations, so, we used our health workers We have some health facilities that don't have much manpower, so we have to still reduce our numbers from that facility to make sure that the vaccination was not only based on fixed post. The outreaches affected the size of manpower at the health facility because the workload was too much. For instance, if there are four (4) staff in a facility and two (2) out of the four (4) go out on outreach then the workload will be much on the remaining two (2) staff (**IDI/Cold Chain Officer/Bosso LGA/Niger State**)*.

(b) *Addressing Health Workforce Challenges*

*Engagement of ad-hoc staff*

However, to address the challenges, partners engaged some teams and paid them stipends for transportation, and weekly completed targets. Additionally, while the state health authorities on the one hand, recruited their volunteers to support health workers going to the community, the partners on the other hand were also responsible for their stipends for their various roles.

*COVID-19 vaccination without mobilization strategy for health workers will amount to nothing. We were able to put appropriate structures in place ensuring that the 80 teams we engaged in the state were a game changer. We had 80 teams for the Sydani teams across the 23 LGAs. We were able to maximize the support and delivery of the teams to ensure that those LGAs or those terrains where there are difficulties in getting people to get vaccinated or where there are challenges of not being able to go there because of limited workfore (**KII/State Service Delivery Focal Person/Sydani*** ***Group**)*.

*Initially, we started by inviting people from the primary healthcare centres and we trained them. We invited trained volunteers and retired health workers to do vaccination and eventually at a later time we had to call teams and that helped to increase the number of workforces you see… so we have about eight teams, in each team we have a minimum of six members and that gives you about forty-eight persons that work with us for vaccination supported by our own technical and leadership team for the state (**KII/Heartland Alliance*** ***Zonal Director**)*.

These key roles played by partners not only positively influenced the workforce but acted as a driver of sustenance among the health workers at the community level. Additionally, the reward system aided the maintenance of commitment on the part of the health workers, and accessibility to vaccine on the part of communal residents.

#### Health information system

##### Dysfunctional electronic reporting

Nigeria has been transitioning from paper-based data reporting to electronic-based data reporting over the past two decades, and this has resulted in the emergence of various health databases or repositories in the country today. At the beginning of the COVID-19 vaccination exercise, states were requested to report their vaccination through two platforms: The National Check-in Call platform, and the Electronic Management of Immunization Data Support (EMID) platform. Before Sydani Group came to Niger and Benue states, there were challenges surrounding the use of the electronic-based data reporting platforms. This hindered the timely reporting of vaccination data that could have informed decision-making. The following excerpts provide more evidence to the discussion:

*Then, there was an issue with the reporting system, initially when they introduced the EMID there were issues. People were unable to upload their reports, and because of that the reports were not sent. And payment was made according to the work done and it looked like they were not working. Also, some teams could not log in with their EMID codes, and this did not let the teams work too (**IDI/State Health Education Officer/Benue*** ***State**)*.

*When they first brought the EMID for our teams to use in reporting, there were issues like not being able to log in, and not having EMID codes. So, we tried to see that it worked but it didn't work. So, we complained to the state, our complaint was mainly to the state and the state tried to fix it but they did not get it (**IDI/LGA M&E Officer/Otukpo/Benue State)***.

##### Improved data reporting systems

To address the issue, one of the partner organizations (Sydani Group) was able to liaise with the national health authority to solve the EMID-related issues that hindered reporting on the part of the mobile teams at the community level. Additionally, partners such as Breakthrough Action Nigeria, RISE, and Sydani Group leveraged existing systems to drive improved reporting structure at the state level and implemented sustainable measures to maintain the improved reporting structure.

*So, another thing we did was invite EMID focal persons, and EMID engineers from the national level to come around, fix these issues, and conduct the capacity-building session with LGA M&E, and state M&E officials. So, that when issues like this come up, they can troubleshoot and fix it for the vaccination teams (**KII/Junior Analyst/Sydani*** ***Group**)*.

*Well, kudos to the state and national leadership. There is already a robust system for data collection and data management. So, what we did was just to see how we could facilitate the periodic reporting of that system, updating that system, so that we are sure that everything has been captured as close to real-time as possible. From our end, we also had like a data management system where we could easily access, see our performance, and report back to the state and ensure harmonization between our data and their data. So, it was more of ensuring data correctness, timeliness, and also supporting the state in areas where they needed help in pushing data upward (**KII/State Service Delivery Focal Person for Benue State/Sydani Group**)*.

The role partners played under the information management system was to improve the extant reporting system that was adopted at the state level from the national level to ensure adequate standard reporting. The partners equally utilize the reporting system to track and monitor the performance of the community teams, the report is shared with the state and the national. The reporting system equally served as a tool for partners to measure the impacts of their support to the states.

#### Finance

Findings from the study participants at different levels show that the financial method adopted by the partners who engaged the mobile teams to drive the optimization of COVID-19 vaccination was rooted in the performance-based finance mechanism.

##### Performance-based financial mechanism

This mechanism was designed to pay health workers (who partook in the activity through the partners) based on the number of people they were able to vaccinate in the various communities. Participants equally empathized that this payment method was very efficient, and as such this strategic financial approach according to the participants could be considered as one of the drivers that improved vaccination coverage in the two states.

*There is a little stipend they give to them weekly because they report weekly. There is a way they calculate it, they will look at the quantity of work you have done and then pay. These stipends are given to them, and if they vaccinate beyond the target, they get a bonus that will be given to them, and their money will be above those that only meet the target. Because of the bonus, so many of them wanted to work more so that they will get the bonus, and by the time they are competing with their mates, and they got this money, you will see that people will be looking at them as the champion for COVID-19 vaccination exercise (**KII/Health System Strengthening Advisor for Benue State/Sydani*** ***Group**)*.

*Well, it helped in terms of boosting the COVID-19 coverage and, it has also helped in reaching the hard-to-reach population because the participants know that they are going to be paid based on coverage, everybody is up and doing. And with this, they will be able to at least go down to the last man within the settlement or the catchment area of their coverage. So, it has helped, and it has also helped the state to get the COVID-19 vaccination down to where we least expected because of the sponsorships (**IDI/State M&E Officer/Niger*** ***State**)*.

*Yes, partners are using what one could call Carrot and stick kind of motivation, for instance, if you place one million naira (# 1,000,000) in front of 10 people and you say the first person to reach where the money is kept will be given one hundred thousand naira (# 100,000), so the payment structure was based on team coverage which was a very strong motive. They set a benchmark of 60 vaccinations on daily basis. So, if you can vaccinate about 120 that is 60 times 2, and the pay for vaccinating 60 people is two thousand naira (#2000) then, the team that vaccinated up to 120 would be paid four thousand. While those who vaccinated between 60 to 100 persons would be paid maybe three thousand. So, this approach has made our teams work extraordinarily, yes, with extra efforts (**IDI/LGA M&E Officer/Ushongo LGA/Benue State**)*.

##### Challenges with payment system

Participants believed the payment mechanism fostered healthy competition among the teams based on the bonus payment included in the payment structure across three categories for teams who can vaccinate beyond the daily target. This subsequently translated to the exponential increase in the number of eligible vaccinated populace. While this was remarkable in itself, suffice to say that participants also identified a few challenges experienced.

*I will say that none of the challenges was man-made but some of the challenges were attributed to technology and some were attributed to systems for example when payments were made initially, they were made from the National primary health care to the state and then, to the LG and from the LG sometimes to the beneficiary. It was discovered that because of the enormity of the team members sometimes when payments are made not all of them receive this at the same time because of the hard-to-reach and rural nature of the majority of the state (**IDI/PM SERICC/Niger*** ***State**)*.

*Generally, one of the challenges that Sydani encountered was an issue with the system in place for the payment. For instance, we had to first confirm the submission of data from the vaccination team, and we also had to know the total number of people that they have vaccinated to determine their pay. And when the teams have issues in the localities with reporting their data, it affects their pay, because we only pay teams based on the data we have. Another thing was that some people made mistakes with the account details they provided, and their payment bounced back, so it caused delays (**KII/Junior Analyst/Sydani*** ***Group**)*.

The challenges associated with the financial mechanism adopted were mostly outside the control of the mechanism implemented by the partners. Technological issues with reporting data and wrong account information were preponderant of other challenges that delayed the payment of the teams in many instances. Nevertheless, the efficiency of the method was proved beyond reasonable doubt to be influential in driving the optimization of vaccination in the two states.

#### Leadership

Participants revealed that before the arrival of partners in the states, there were existing leadership structures at the state and LGA levels, and partners leveraged this structure to ensure accountability and transparency in the optimization of COVID-19 vaccination. Partners such as Sydani Group engaged the state and LGA levels health officials for supervisory roles to monitor and track the teams, and address on–the–field challenges experienced by the mobile teams.

*During the implementation, I will say everything lies in the cold chain office, but we have our director there. Everything we did during that period when we received the vaccine we have to tell our director the number of vaccines then by telling the director we tell the team everybody would come for the vaccine. When they receive that vaccine when we go for vaccination we made sure that the director, LIO, CCO, the RIO, and the M&E will go for supervision and if there is any problem during the vaccination period, they will make sure to report to the LIO or the CCO then we will now forward the issue to the director (**IDI/Cold Chain Officer/Bosso LGA/Niger*** ***State**)*.

*What we utilized was the existing leadership structure in the state. So, we engaged LGA supervisors to provide that level of closed supervision to the vaccination teams across all 23 LGAs of implementation. And then we also engaged the state supervisors to provide that top or higher level of support, that is, supportive supervision. We also engaged independent supervisors to supervise LGAs we could not go. So, yeah, it was that sort of hands-on supportive supervision across the existing structures. And then at some point we included, or we came up with our strategy for supportive supervision (**KII/Senior Analyst/Sydani*** ***Group**)*

*Yes, what they did, I think especially the Sydani Group, they called the LGA teams into training at Bida, of which me and my CCO, director, M&E health educator, we went and were imparted with this knowledge. So, when we come back, we put to work the knowledge we have received, we also cascade the training to other health workers. and supervise their work. We divide the LGA into teams (**IDI/LGA Immunization Officer/Edatti LGA/Niger State**)*.

Evidence from the interviews echoed the leadership structures across the LGA and state levels. It equally suggests that when these key actors at the LGA and state levels (such as the SIO, LIO, and M&E) were engaged in the supervisory role, they were motivated by the stipends they received to sustain the reporting system, which subsequently translated into improved vaccination numbers reporting among the mobile teams. While supervision was being conducted by stakeholders within the system, partners and/or engaged independent supervisors also took on supervisory responsibilities to drive the successful operation of optimizing COVID-19 vaccination in both states.

### Participants perception toward partners' supports in Benue and Niger states

#### Responses from state and local government actors

Interestingly, participants from the states and LGAs find the support offered to the states significantly influential to the optimization of COVID-19 vaccination. The participants emphasized that monetary motivation offered to the teams by the partners was the major driver of the improved vaccination coverage that both states experienced. Participants equally expressed that the support was impactful in that it elevated their positions in the national ranking on COVID-19 vaccination coverage.

*I would describe it as excellent because it has pushed us to where we are expected to be. When we started COVID-19 we were performing well but later we were not doing well. I think among the ten least performing states in the nation but when the partners came in, we kept on pushing we became let's say 13th position and we were improving gradually. So, I think by now we're among the first ten states in terms of coverage. Because now we're more than 80 percent coverage based on the national target, and all these we achieved as a state through the help of all these partners that actually came in to support our teams (**IDI/State Immunization Officer/Niger*** ***State**)*.

*Well the partners have done a very good job you understand because by supporting the teams they have done a great job that make people have access to the vaccine, I must say the truth you understand, because without the partners giving money to the teams they will not be able to go out and vaccinate those people because some people cannot even come out of their places and they have to go to those places to vaccinate them…so at that area, they have done a great job (**IDI/Cold Chain Officer/Otukpo LGA/Benue State**)*.

#### Implementing partners response

While stakeholders at the state and LGA levels remained enthusiastic about the attained milestone, it was equally important to understand the perception of the partners on their support to the states. The partners, however, shared similar euphoric satisfaction from the outcome of the support provided to the states.

*Well, we have great influence on COVID-19 optimization in the state which has played a significant role in the state. Our support helped the state to understand the vaccine distribution and the required areas to focus on in Niger State. Just to also mention that, before the vaccination, there were no structured target areas or communities. I think it was when the Sydani team came in for another phase of support that there were targeted populations across areas in Niger state due to our support which enlightened the team on the coverage in the state (**KII/Strategic Information*** ***Associate/Palladium**)*.

*I think it's significant if you look at the data and the good number compared to when the state started noticing the number. They were aware that something had changed, they could see the impact. I believe that our contribution is significant, and it has helped the state in the way they plan and anticipate outcomes (**KII/Heartland Alliance Zonal Director/Niger*** ***State**)*.

*We have tried our best and the state has been able to record some significant improvement over the past few months. And it's a collaborative effort. All available partners have contributed (**KII/WHO State Representative/Benue State**)*.

Partners were also impressed by the quantum leap the state experienced through the support provided to the state and local government health authorities to optimize COVID-19 vaccination. However, partners emphasized that the collaborative efforts between partners played a pivotal role in the strategies developed to drive optimization. Additionally, the collaborated efforts between the partners, and state and local government health authorities were significantly integral to the shared success story of all the players involved.

## Discussion

The study was initiated to explore the intersection between partnership coordination and the optimization of COVID-19 vaccination in Benue and Niger states. Our study is premised on filling the gap in the assessment of stakeholders' (partners) roles in the optimization of COVID-19 vaccination in Benue and Niger states. For structural exploration, we divided the result sections into three segments. First, we assessed the perception of stakeholders in both states across the state and LGA levels on the reality of the COVID-19 vaccination exercise over the last 2 years (from the inception of vaccination in the states, hitherto). Secondly, having adapted the WHO Health System Building Blocks Framework, our study investigated the contributions of various local and international partners to strengthening the health systems, especially the immunization unit, in each state, using five thematic areas. Finally, we sought all involved participants' perceptions on the influence of support to the states on the optimization of COVID-19 vaccination in both states.

The demographic component of our study revealed more males were represented than females in the study. This disparity is premised on the gender imbalance in the positions of health administrator in the selected states identified for the study. This gender gap does not in any way has implication on the study findings, as the focus of our study is on exploring the reality of partnership coordination and it's influence on the optimization of COVID-19 vaccination in the selected states. Findings from the study revealed that at the onset of COVID-19 vaccination in the states, considerably after the strategies adopted by the state health authority to vaccinate their respective populaces with COVID-19 vaccines, there remained a low–level of awareness as regards COVID-19 vaccination in the state. This challenge may be fostered by the limited creation of awareness on COVID-19 vaccination, misinformation, and conspiracy theories that pervaded most low and middle-income countries, especially in semi-urban, and rural settlements about immunization services, and COVID-19 vaccination in particular.

Findings from the study of Womodi et al. (20) revealed that participants from different communities across the country have heard one or more conspiracy theories about COVID-19, the validity of the pandemic response, and COVID-19 vaccines. The study also exuded that there existed limited accessibility of COVID-19 vaccines to residents in Benue and Niger states in the first few months of COVID-19 administration in both states. This may be premised on conditions outside the control of the health authorities including security issues, environmental challenges, and structural problems. This alludes to the study of (21) who found security challenges as contributing factors to the limitation of COVID-19 vaccination coverage among health professionals in Somalia. The study equally found that there was limited coverage of the COVID-19 vaccination in both states at the initial phase of the COVID-19 vaccination exercise. The limited coverage may be influenced by a culminating push and pull factors of vaccination barriers in the states such as vaccine hesitancy, limited education, logistics, unavailability of vaccines, poor logistics, and so on. This is similar to the findings of Bergen et al. (22), who reported structural barriers and vaccine hesitancy as drivers of low vaccination coverage in several low and middle-income countries.

Subsequently, our study findings revealed that Benue and Niger states established partnerships and accessed supports during COVID-19 vaccination exercise from various local and international donor organizations such as United Nations International Children Emergency Funds (UNICEF), World Health Organization (WHO), and implementing partners such as Sydani Group, Breakthrough Action Nigeria, Heartland Alliance, and Koninklijke Nederlandse Chemische Vereniging (KNCV) Tuberculosis Foundation. Based on the study findings, the partners supported the states to address extant challenges identified in the optimization of COVID-19 vaccination in both states. By implication, there is an improvement in the availability of resources at the disposal of health administrators in Benue and Niger state. Various forms of collaboration were operated globally to provide support LMICs (23, 24), and was established to have improved the availability and administration of COVID-19 vaccine. For instance, Soni et al. (25) found that the synergetic collaboration among governmental, non-governmental organization, and at risk vulnerable communities in India, led to the success of reaching nearly 50 million in messaging, and getting almost one-third of that population vaccinated.

Additionally, the partners provided support on different thematic areas in promoting and optimizing COVID-19 vaccination in each of the two states, and in strengthening the health systems of both states. This is in tandem with the support of the COVID-19 Vaccine Global Access Facility (COVAX) provided to the Nigerian government and National Health Authorities with the provision of 115 million doses of COVID-19 vaccines (26).

Furthermore, findings from our study revealed that in the area of service delivery as a thematic focus, partners in both Benue and Niger states played significant roles that culminated in tremendous efforts geared toward quality service delivery of COVID-19 vaccine in both states. The study results also show that partners were highly intentional in ensuring that the states met their target through improved coverage rates in various localities of both states. Specifically, one of the participants emphasized that to drive evidence-based service delivery methods, stakeholders across different levels were onboarded and/or trained in their respective roles to ensure that all parties involved meet expectations.

Additionally, participants emphasized that the monetary motivation offered to mobile teams to ensure door-to-door campaigns and vaccine administration was an important driver of improved service delivery. The provision of SDDs is highly commendable as it proved its worth through cost reduction while improving the availability of vaccines. Similarly, strategies were put in place to ensure that there was improved service delivery, and this was done through the combination of multiple approaches, and a collaborative partnership effort in the state.

This alludes to the work of Soeters et al. (27) who asserted that the US-CDC has contributed to the development of global technical tools, guidance, and policy for COVID-19 vaccination through the support offered to LMICs' health authorities and their partner organizations, using seven priority areas of technical assistance. In a further breakdown, findings indicated that partners' provision of funds was preponderantly the contributory efforts to ensure adequate quality service delivery in the states, albeit other forms of support such as technical support, and vaccine logistics were offered to the states toward ensuring adequate quality service delivery to the residents of Benue and Niger states. These supports were used to drive public awareness of COVID-19 vaccination, organize capacity-building sessions for implementers across the state and LGA levels, as well as to provide logistics for vaccines, and the mobilization of health workers visiting communities to educate and vaccinate community residents. Partners also provided a Solar Direct Drive (SDD) refrigerator to several facilities which helped to ensure the safety and maintained good potency of the COVID-19 vaccines.

On the health workforce, findings from our study revealed that before the advent of partners in the state, health workers administering vaccines in Benue and Niger states were not adequately renumerated, and the strength of the workforce was considered insufficient by the participants. These issues may be premised on the inadequate funding of the health sector by the federal and state governments in Nigeria. This established the findings of Olateju et al. (28), who reported that the provision of financial incentives alleviated most of the challenges associated with the health workforce deliverables in their study area. Consequently, the partner's involvement in the states, and through their supportive contribution to the optimization of COVID-19 vaccination in the states, facilitated the weekly, and monthly remuneration of the health workers, which significantly influenced their morale, especially in administering vaccines to the populace across various communities in their respective LGAs. Findings also revealed that partners such as Sydani Group engaged more mobile teams to ensure the optimization of COVID-19 vaccination in Benue and Niger states.

Additionally, findings from our study revealed that at the state and LGA levels in Benue state, stakeholders received complaints from the teams regarding the challenge of reporting COVID-19 vaccination on the EMID platform at the initial phase of COVID-19 vaccination, which subsequently affected the reporting rates of the state even though the mobile teams were able to reach a lot of the residents and get them vaccinated in various communities. Our study found that partners played a key role in addressing the problem by bringing in a focal person from the national level to address the challenge. Partners also leveraged the opportunity to have the state and LGA representatives trained to ensure a sustainable reporting system on vaccination services.

Subsequently, our study findings revealed that partners such as Sydani Group, KNCV, and RISE, who remunerated mobile teams with stipends for logistics and motivation for driving COVID-19 vaccination exercise adopted a Performance-based financial mechanisms where health workers were paid on an assigned daily target, and their payments were subsequently increased when the team exceeded their daily targets. Although payments were relatively made to the team by the respective organizations, our study participants unanimously emphasized that the payment strategy adopted was significantly integral to the success of COVID-19 vaccination optimization in Benue and Niger states (attaining the national targets), as the strategy was observed to have created competition amongst the teams, yielding ripple effects on vaccination coverage in the various communities of the two states. This alludes to the recommendation of Mushasha and El Bcheraoui (29), that funding agencies may achieve better outcomes from their investment by providing a results-based financing system that combines demand and supply sides health-related schemes.

Furthermore, our study found that partners leveraged existing leadership structures at the state and LGA levels to establish accountability and transparency in the operation of the COVID-19 vaccination exercise in Benue and Niger states. Essentially, our findings revealed that health officials at the LGA level were engaged by Sydani Group to conduct supervision thrice weekly using a computer-assisted tool (Kobo Collect) to confirm their activities and were reimbursed for their transport by the partners. Additionally, health workers at the state levels were equally engaged by Sydani Group to conduct supervision two times a week at the community and LGA levels to ensure adequate and quality data reporting and vaccination exercise in their respective states.

Finally, findings from our study revealed that health officials at the state and LGA levels in Benue and Niger states found the support provided by partners to each state to optimize COVID-19 vaccination, to be excellently remarkable in terms of the milestone achievements, support offered to the states, as both states attained and exceeded the desired national targets of 70%. Findings also revealed that partners were impressed to have helped the states, through concerted efforts attain a quantum leap in vaccinating their residents, thereby establishing herd immunity in their respective states, and improving the health of the population in both states.

## Limitations of the study

A potential limitation to this study is the social desirability bias, as the study participants might have responded to questions in a socially acceptable or desirable manner, rather than the reality in their respective state. Although, the study was designed to include two local government areas per senatorial district, security challenges reported in the state across some LGAs (including two of our initially selected LGAs) at the time of the study influenced the replacement of two LGAs in our study, resulting in a skewed LGA selection process. The geographic location of the study sites (two north central states) limits the extent to which the stud findings can be generalized. Additionally, findings from the study may not be generalizable particularly because of the study sample size and sampling technique, owing to the subjective nature of the unit selection. Furthermore, the engagement of more male stakeholders (government and partners) over female stakeholders for the various roles considered for the study limited the participation of females in this study. Finally, this study did not explore the challenges and limitations of collaboration, and this area can be subsequently researched upon.

Consequently, two limitations of this study will have a little impact on our study findings. First, the lack of generalizability of qualitative study limits the potential impact this study's findings may have on the larger low and middle income countries, though they can still take learning from this. Finally, the inadvertent omission on exploring the challenges experienced by all stakeholders in the process of collaboration will limit the level of available robust information on the subject matter.

## Conclusion

Similar to other sectors outside health, private-public partnerships have been established numerously to achieve one or more goals, and these on many occasions have always resulted in success stories of various types. Although international agencies and donors of categories are renowned for providing technical and funding support to the government (at the national and sub-national levels) of low- and middle-income countries, aimed at improving the health of their population, especially the vulnerable groups, such as women, children, and older adults, and sometimes, the support is geared toward the entire population. These supports elucidate the importance of pooling resources (funds or expertise) together to achieve a common goal, as in this case, driving COVID-19 vaccination uptake in Benue and Niger states, Nigeria.

These supports are usually utilized to design approaches to addressing health challenges and implications, particularly around issues of healthcare accessibility and inequities. However, our study has been able to establish that support can also come in the form of partnerships to promote the health of the population in a given community. As such this study finds it plausible to have a government at the national and sub-national levels establish a positive relationship with international and local organizations as well as implementers in co-developing a comprehensive health strategy to create solutions to manage and eradicate fundamental public health concerns that pervade such country, region, and state and thereby making quantum leaps toward the attainment of universal health coverage.

Specifically, the study recommends the establishment and/or re-engineering of partnership platforms within the health industry in the state, where biannual gap analysis are conducted in the state health systems to identify areas that require improvement through the provision of support. The partnership platforms can also conduct a resource mapping of support provided to the state, particularly in regard to the focus areas of partners in the state and the capacity (technical or financial) in which the support is provided to the state. This helps the states to have a systematic process in place to easily identify gaps and areas that require improvement within the state health systems. This also sets precedent for cases of emergency relating to the state residents' health.

## Data Availability

The original contributions presented in the study are included in the article/supplementary material, further inquiries can be directed to the corresponding author.
